# Fellowships, Grants, & Awards

**Published:** 2005-07

## Stem Cells and Cancer

The clonal nature of most malignant tumors is well established. Experiments spanning several decades have shown, however, that as many as one million murine or human tumor cells are required to transplant a new tumor from an existing one. Two theories have been developed to account for the observation that apparently not every tumor cell is a tumor initiating cell (T-IC). The stochastic theory predicts that every tumor cell can form an entirely new tumor; however, entry into the cell cycle is a stochastic event with low probability. Alternatively, tumor cells may exist in a hierarchical state in which only a small number of cells possess tumor initiating potential. If the stochastic model is correct, then tumor cells are biologically homogeneous and genetic or epigenetic programs that allow for tumorigenesis are operative in the majority of cells that comprise a tumor. The hierarchical model, however, predicts the tumor cells possess a functional heterogeneity and that quantitatively the cells capable of tumorigenesis are a relatively minor population among the bulk of tumor cells.

Recent data from both hematologic malignancies and solid tumors have suggested that there are only minor populations of cells in each malignancy that are capable of tumor initiation. These T-ICs have the functional properties of tumor stem cells. They appear to be capable of asymmetric division and self renewal, are only a minor faction among the bulk of more differentiated cells in the tumor, and can reconstitute all the cell types in the tumor of origin.

Currently, tumor stem cells have been isolated and characterized in several hematologic malignancies and some solid tumors. One of the first tumors in which a stem cell was identified was acute myeloid leukemia (AML). In this disease, the frequency of the leukemic stem cell (LSC) was approximately 1 per million AML blasts, establishing that not every AML cell had T-IC capacity. A CD34 positive/CD38 negative cell fraction representing 0.1 to 1 percent of the tumor cells possessed all the leukemia initiating activity in the NOD/SCID transplantation model. By contrast, the major fraction of the CD34 positive/ CD38 positive cells and the majority of CD34 negative cells, which comprise most of the cells in the tumor, could not initiate leukemia. A multiple myeloma stem cell has also been characterized. Multiple myeloma cell lines and primary patient derived cells express the cell surface marker syndecan-1 (CD138). A population of cells representing less than 5 percent of the cells in the bulk population of multiple myeloma cells was found to be CD138 negative, and possessed *in vitro* clonogenic potential. These cells also engrafted successfully into Non-obese Diabetic/Severe Combined Immunodeficient (NOD/SCID mice), whereas CD138 positive cells did not engraft.

Recently, a mammary carcinoma stem cell has been isolated from primary mammary carcinomas using four cell surface markers (CD44; CD24; a mammary tumor marker, and epithelial specific antigen). The tumor initiating capacity of the cells was again verified in an *in vivo* NOD/SCID engraftment assay. The mammary tumor stem cells represented only 2 percent of the unfractionated bulk tumor cells.

Finally, a putative brain tumor stem cell has also been isolated. These cells appear to be between 0.3 to 25 percent of the cells in the brain tumors examined. They are positive for the neural stem cell marker CD133 and have a marked capacity for self renewal and differentiation. Transplantation of these putative neural tumor stem cells into the forebrains of NOD/SCID mice yields tumors phenotypically resembling the tumors from which the stem cells were isolated.

Isolation of tumor stem cells from a larger spectrum of solid tumors and characterization of markers for such cells will be important in understanding how general the role of tumor stem cells are in the pathogenesis of cancer.

In addition to isolating and characterizing tumor stem cells themselves, it is also important to identify the genes and proteins that facilitate the self renewal phenotype that characterize all stem cells. The proteins involved in self renewal in embryonic and adult stem cells appear to be subverted in tumorigenesis to allow the tumor-initiating cells to maintain self renewal capacity. Two families of proteins related to self renewal, the polycomb gene Bmi-1 and the Wnt signaling pathway proteins, have been related to the maintenance of the tumor stem cell phenotype.

The polycomb genes have an essential role in embryogenesis, regulation of the cell cycle, and lymphopoieisis. These genes are transcriptional repressors that are essential for the silencing of other families of genes. Deletion of polycomb gene Bmi-1 in mice results in a progressive loss of all hematopoietic lineages. This loss results from the inability of the Bmi-1(−/ −) stem cells to self renew. Introducing genes known to produce AML into Bmi-1(−/ −) hematopoeitic stem cells (fetal liver cells), induced AML with normal kinetics; however, the Bmi-1(−/ −) leukemic stem cells from primary recipients were unable to produce AML in secondary recipients. These results demonstrate that Bmi-1 is also required for self renewal of leukemic stem cells in the murine AML model.

Another group of genes involved in self renewal are those involved in the Wnt signal transduction cascade. The Wnt protein binds to a receptor called Frizzled and activates cell fate decisions during tissue development. Inhibitors of Wnt signaling produce inhibition of hematopoietic stem cell growth *in vitro* and reduce hematopoietic reconstitution *in vitro*. Activation of Wnt signaling in hematopoeitic stem cells leads to increased expression of Hox B4 and Notch-1 genes previously implicated in self renewal of hematopoietic stem cells. The Wnt signaling pathway is involved in both hematopoietic malignancy and colon carcinoma. Although the Wnt ligands themselves are only rarely involved in tumorigenesis, mutations mimicking Wnt receptor (Frizzled) activation induce a set of genes associated with repression of differentiation and potentiation of self-renewal. In general, these mutations involve Wnt signal transduction proteins including the activation of beta-catenin and inactivation of the (APC) adenomatosis polyposis coli protein. In myeloid leukemia, nonphosphorylated beta-catenin accumulates in granulocyte-macrophage progenitors as they progress toward leukemia. These normally more committed progenitors can thus acquire self-renewal properties. A similar accumulation of unphosphorylated beta-catenin has also been observed in multiple myeloma cells. In colon cancer, the APC gene is mutated early in the development of about 90 percent of the colon carcinomas. Similarity in gene expression patterns between populations of colon cancer cells and colon epithelial stem cells has also been observed by DNA microarray analysis. It is possible that mutations in the Wnt signaling pathway maintain the program of stem cell genes in the transcriptionally active state.

Additional research on the important genes and proteins that function to maintain the stem cell phenotype could contribute to increasing the number of specific targets for which cancer therapeutic agents can be designed.

Recent studies of Helicobacter infection in mice have suggested that bone marrow hematopoietic stem cells can contribute to repopulating the gastric mucosal epithelium. These cells can progress, with time, to metaplasia, dysplasia, and cancer. The possibility of stable fusion between the bone marrow derived cells and the gastric mucosa was eliminated, suggesting the possibility of transdifferentiation of hematopoietic stem cells. Research aimed at establishing whether or not stem cells have the capacity to transdifferentiate is also encouraged.

This funding opportunity is intended to promote research on all aspects of tumor stem cell biology, and on the genes and proteins responsible for the tumor stem cell phenotype. Research studies on the characterization of tumor stem cells from the broad spectrum of solid and liquid tumors not already examined, on markers potentially shared by tumor stem cells and normal stem cells, and on the biochemical and molecular regulation of normal and tumor stem cell function are encouraged. Such research can and should include research on *in vivo* assays for the functional identification of such cells. Studies of the genes regulating self renewal, and studies of regulation of stem cell division by the stem cell niche and/or microenvironment are also encouraged. Investigators working on the cell and molecular biology of embryonic stem cells, adult stem cells, and tumor stem cells are encouraged to apply for support under this funding opportunity. The following questions illustrate areas of high interest, but other relevant innovative projects are also encouraged: 1) What governs the proliferation rate of normal and tumor stem cells? 2) Can oncogenes and their associated mutations affect asymmetric versus symmetric divisions in stem cells? 3) Stem cell quiescence versus growth must ultimately be understood in terms of progression through the cell cycle. Which stem cell-specific genes alter the cell cycle pathway proteins? 4) Do tumor stromal cells constitute a unique tumor stem cell niche? Does the tumor stromal niche act as a constituent of a feedback mechanism with tumor stem cells to control their growth? 5) Are the phenotypes of invasion and metastasis uniquely connected to the tumor stem cell phenotype? 6) Are normal resident adult tissue stem cells a special target for carcinogenic insults? 7) Can new and/or better markers and assays for the isolation and enrichment of tumor stem cells be developed? 8) Can new and/or better *in vivo* functional assays to identify tumor initiating cells (e.g., engraftment of leukemic stem cells into immunodeficient NOD/SCID mice) be developed? 9) How do changes to stem cells or their environment due to aging affect formation of tumor stem cells or alter their properties?

This funding opportunity will use the R01 and R21 award mechanisms. As an applicant, you will be solely responsible for planning, directing, and executing the proposed project.

This funding opportunity uses just-in-time concepts. It also uses the modular as well as the non-modular budget formats (see http://grants.nih.gov/grants/funding/modular/modular.htm). Specifically, if you are submitting an application with direct costs in each year of $250,000 or less, use the modular budget format described in the PHS 398 application instructions. Otherwise, follow the instructions for non-modular research grant applications.

The PHS 398 application instructions are available at http://grants.nih.gov/grants/funding/phs398/phs398.html in an interactive format. Applicants must use the currently approved version of the PHS 398. For further assistance, contact GrantsInfo at 301-435-0714, (telecommunications for the bearing impared: TTY 301-451-0088) or by e-mail: GrantsInfo@nih.gov.

Applications must be prepared using the most current PHS 398 research grant application instructions and forms. Applications must have a D&B Data Universal Numbering System (DUNS) number as the universal identifier when applying for Federal grants or cooperative agreements. The D&B number can be obtained by calling 866-705-5711 or through the web site at http://www.dnb.com/us/. The D&B number should be entered on line 11 of the face page of the PHS 398 form. The complete version of this PA is available at http://grants/guide/pa-files/PAR-05-086.html.

Contact: R. Allan Mufson, Cancer Immunology Hematology Branch, Division of Cancer Biology, National Cancer Institute, 6130 Executive Blvd, EPN Rm 5062, Bethesda, MD 20892 USA, Rockville, MD 20852 USA (for express/courier service) 301-496-7815, Fax: 301-480-2844, E-mail: am214t@nih.gov; Jill Carrington, Systems Branch, Musculoskeletal Biology, Biology of Aging Program, National Institute on Aging, National Institutes of Health, 7201 Wisconsin Ave, Suite 2C231, MSC 9205, Bethesda, MD 20892 USA, 301-496-6402, fax: 301-402-0010, e-mail: carringtonj@nia.nih.gov. Reference: PA No. PA-05-086

## Secondary Analyses in Obesity, Diabetes, Digestive and Kidney Diseases

The specific objectives of this announcement on Secondary Analyses In Obesity, Diabetes, Digestive And Kidney Diseases are to support the following: (a) research on secondary analyses of data related to the epidemiology of disease areas of NIDDK; (b) preliminary projects using secondary analysis that could lead to subsequent applications for individual research awards; (c) rapid analyses of new databases and experimental modules to inform the design and content of future studies; (d) the archiving of data sets to be made publicly available for research purposes related to disease areas of NIDDK, including both epidemiological studies and multi-center clinical trials.

Research that employs analytic techniques that demonstrate or promote methodological advances in patient-oriented and epidemiologic research is also of interest. International comparative analyses are encouraged. Applications that are innovative and high risk with the likelihood for high impact would be especially encouraged.

Patient-oriented and epidemiologic research projects, particularly multicenter projects, typically generate data with potential utility beyond the specific hypotheses and questions for which they were designed. Often data are not fully analyzed, especially when unexpected research questions emerge after the end of the project's funding period. Analyzing such existing data sets can therefore provide a cost-effective means to test specific hypotheses that have not been adequately examined. The further analysis of existing research data may also be prompted by a need to confirm new findings or to aid in the development of new research questions.

Applicants may conduct secondary analyses using data from a variety of sources. These would include investigator-initiated research activities, cooperative agreements, and contracts or from other public or private sources. Sources may be large, nationally representative data sets such as those of the National Center for Health Statistics or smaller, regional or locally based data sets. Also appropriate for secondary analyses are relevant cross-sectional and longitudinal survey data collected by federal, state, and local government agencies. Secondary data analyses of these data may serve as an economical alternative to expensive and time-consuming new data collection projects. Applicants may also secure access to other data sets that may or may not be in the public domain, such as those collected under research grant funds, sponsored by private entities, or originally collected for purposes other than research, such as health care administrative data sets.

In addition to the examination of specific research hypotheses, existing data sets may also be used to cross-validate exploratory analyses in ongoing studies, to test complex statistical models, and in special circumstances to provide comparison groups for experimental studies. Moreover, secondary analysis is appropriate for many types of data, including qualitative information, and may also cover the integration of quantitative and qualitative data.

NIDDK has established a repository for the archiving of data sets, as well as genetics and tissues from NIDDK sponsored clinical trials and epidemiological studies (http://pubnts06.rti.org/niddk/home.do). Applicants are encouraged to consider the research opportunities available in this NIDDK resource.

A major interest of NIDDK is supporting secondary data analyses in the causes, burden, natural history, and treatment and medical care of overweight and obesity, including analyses of behavioral/environmental factors that may be predictive of long term weight maintenance or prevention of weight gain. Other specific subject areas are restricted to those on which NIDDK conducts research, which include diabetes and endocrine and metabolic diseases; digestive diseases and nutritional disorders, including eating disorders; and kidney, urological, and hematological diseases. All data analyses must concern patient-oriented or epidemiologic research designed to elucidate the etiology, incidence, prevalence, natural history, pathophysiology, or response to therapy of the above-mentioned disorders.

This program announcement addresses several areas considered to be high priority for liver disease research as delineated in the recently published Trans-NIH Action Plan for Liver Disease Research (http://liverplan.niddk.nih.gov), specifically in the areas of fatty liver disease, viral hepatitis, drug- and toxicant-induced liver disease, autoimmune liver disease, pediatric liver disease, liver transplantation, complications of liver disease, and gallbladder and biliary disease.

This mechanism can also be used for the merging of secondary data sets with other data sets. For example, if allowed by informed consent, databases could be matched with hospital data sets or vital statistics to assess longer-term morbidity and/or mortality of patient groups. Meta-analysis, in which results from multiple studies may be compared or combined, is encouraged only if patient level data from the original studies are combined.

Support for the creation of publicly archived data sets involved in secondary analyses would be for information that could be applied to the subject areas of interest. Plans for archiving must include adequate dataset documentation and explanation so that it can be used by researchers not associated with the original study. Provision for easy accessibility of archived datasets is required.

Up to 25% of the direct costs of the grant may be spent on acquiring new information that would be incorporated into the database and would significantly strengthen the analysis. Such information may, for example, be derived from laboratory testing of stored specimens or comparisons of a measurement against a criterion standard to validate the measurement in the database. Conversion of data from paper records to electronic form would also be considered up to the 25% limit. Obtaining such new information must serve the purpose of the secondary data analysis and should not be considered for any other reason.

This funding opportunity will use the NIH Exploratory/Developmental (R21) award mechanism. As an applicant, you will be solely responsible for planning, directing, and executing the proposed project.

This funding opportunity uses just-in-time concepts. It also uses the modular budget format described in the PHS 398 application instructions (see http://grants.nih.gov/grants/funding/modular/modular.htm).

The PHS 398 application instructions are available at http://grants.nih.gov/grants/funding/phs398/phs398.html in an interactive format. Applicants must use the currently approved version of the PHS 398. For further assistance contact GrantsInfo at 301-435-0714 (telecommunications for the hearing impaired: TTY 301-451-0088) or by e-mail: GrantsInfo@nih.gov.

Applications must be prepared using the most current PHS 398 research grant application instructions and forms. Applications must have a D&B Data Universal Numbering System (DUNS) number as the universal identifier when applying for Federal grants or cooperative agreements. The D&B number can be obtained by calling 866-705-5711 or through the web site at http://www.dnb.com/us/. The D&B number should be entered on line 11 of the face page of the PHS 398 form. The complete version of this PA is available at http://grants.nih.gov/grants/guide/pa-files/PA-05-091.html.

Contact: James E. Everhart, Epidemiology and Clinical Trials Branch, Division of Digestive Diseases and Nutrition, National Institute of Diabetes and Digestive and Kidney Diseases, 6707 Democracy Blvd, Rm 655, Bethesda MD 20892-5450 USA, 301-594-8878, fax: 301-480-8300, e-mail: je17g@nih.gov; Catherine C. Cowie, Type 1 Diabetes Clinical Trials Program, Division of Diabetes, Endocrinology, and Metabolism, National Institute of Diabetes and Digestive and Kidney Diseases, 6707 Democracy Blvd, Rm 691, Bethesda, MD 20892-5460 USA, 301-594-8804, fax: 301-480-3503, e-mail: cc68v@nih.gov; Paul W. Eggers, Epidemiology and U.S. Renal Data System, Division of Kidney, Urologic, and Hematologic Diseases, National Institute of Diabetes and Digestive and Kidney Diseases, 6707 Democracy Blvd, Rm 615, Bethesda, MD 20892-5458 USA, 301 594-8305, fax: 301-480-3510, e-mail: pe39h@nih.gov. Reference: PA No. PA-05-091

